# FGF2 antagonizes aberrant TGFβ regulation of tropomyosin: role for posterior capsule opacity

**DOI:** 10.1111/jcmm.13030

**Published:** 2016-12-15

**Authors:** Eri Kubo, Shinsuke Shibata, Teppei Shibata, Etsuko Kiyokawa, Hiroshi Sasaki, Dhirendra P. Singh

**Affiliations:** ^1^ Department of OphthalmologyKanazawa Medical UniversityUchinadaKahoku‐gunIshikawaJapan; ^2^ Department of Oncogenic PathologyKanazawa Medical UniversityUchinadaKahoku‐gunIshikawaJapan; ^3^ Department of Ophthalmology and Visual SciencesUniversity of Nebraska Medical CenterOmahaNEUSA

**Keywords:** epithelial–mesenchymal transition, tropomyosin, lens epithelial cells, FGF2, TGFβ2

## Abstract

Transforming growth factor (TGF) β2 and fibroblast growth factor (FGF) 2 are involved in regulation of posterior capsule opacification (PCO) and other processes of epithelial–mesenchymal transition (EMT) such as cancer progression, wound healing and tissue fibrosis as well as normal embryonic development. We previously used an *in vivo* rodent PCO model to show the expression of tropomyosin (Tpm) 1/2 was aberrantly up‐regulated in remodelling the actin cytoskeleton during EMT. In this *in vitro* study, we show the Tpms family of cytoskeleton proteins are involved in regulating and stabilizing actin microfilaments (F‐actin) and are induced by TGFβ2 during EMT in lens epithelial cells (LECs). Importantly, we found TGFβ2 and FGF2 played contrasting roles. Stress fibre formation and up‐regulation of α‐smooth muscle actin (αSMA) induced by TGFβ2 could be reversed by Tpm1/2 knock‐down by siRNA. Expression of Tpm1/2 and stress fibre formation induced by TGFβ2 could be reversed by FGF2. Furthermore, FGF2 delivery to TGFβ‐treated LECs perturbed EMT by reactivating the mitogen‐activated protein kinase (MAPK)/ extracellular signal‐regulated kinase (ERK) pathway and subsequently enhanced EMT. Conversely, MEK inhibitor (PD98059) abated the FGF2‐mediated Tpm1/2 and αSMA suppression. However, we found that normal LECs which underwent EMT showed enhanced migration in response to combined TGFβ and FGF2 stimulation. These findings may help clarify the mechanism reprogramming the actin cytoskeleton during morphogenetic EMT cell proliferation and fibre regeneration in PCO. We propose that understanding the physiological link between levels of FGF2, Tpm1/2 expression and TGFβs‐driven EMT orchestration may provide clue(s) to develop therapeutic strategies to treat PCO based on Tpm1/2.

## Introduction

Age‐related cataract, a chronic disorder of ageing, is the main cause of blindness worldwide. PCO is a common, significant complication following cataract surgery. Advances in surgical techniques, intraocular lens materials and designs have reduced the PCO rate, but it remains a significant problem worldwide, even in young and infant patients 1, 2. After cataract surgery, aberrant cell growth across the lens capsule often leads to fibrosis and secondary visual loss, known as PCO, so‐called secondary or after cataracts 3. EMT of LECs is the main cause 4, 5, 6, 7. EMT is related to other eye diseases such as pterygium and glaucoma and the wound healing process after eye surgery 8, 9, 10. To regulate EMT is important for treatment of many eye diseases including PCO. The current treatment of PCO is YAG laser capsulotomy. However, this method can lead to uveitis, cystoid maculae oedema, elevation of intraocular pressure, retinal detachment and intraocular lens damage. Further, in paediatric cataract, it is clear that cataract extraction and correction of aphakia should be performed as soon as possible during the key period of vision 11. However, PCO is a common complication of the surgery and also leads to amblyopia. YAG laser capsulotomy is not difficult to perform, but it needs patients’ cooperation and eye fixation 11. Secondary capsulotomy surgery is sometimes required in children 12. We believe that it is important to study on the prevention of PCO for child patients. Further, it is important to regulate EMT and PCO in LECs for the clinical treatments using accommodative lens refilling^13^ and for the regeneration of clear lens *in vivo* in future.

Aberrant TGF β signalling plays a central role in the pathobiology of cells or tissues by dysregulating extracellular matrix (ECM)‐related genes in LECs, akin to the development of human anterior subcapsular cataract 14, 15, 16, 17 and PCO 5, 17, 18, 19, 20. Moreover, TGFβs are involved in induction of tissue fibrosis, myofibroblast formation and apoptosis 21, 22, 23 by up‐regulating genes encoding ECM proteins including αSMA, types I and III collagens. Previous studies suggest FGF may contribute to PCO development. FGF2 is expressed in human LECs 24 and involved in lens development 25 regulating cell proliferation and migration. This molecule is involved in stimulation of lens fibre differentiation in a dose‐dependent manner 26, 27 and also activates LEC mitosis increasing the formation of collagen 28. FGF2 was shown to reduce the contraction of a collagen gel in bovine LECs and the proportion of cells expressing αSMA 29, indicating that FGF2 acts contrary to TGFβ. Moreover, EMT in PCO was reported to be regulated by ECM components and soluble growth factors or cytokines, including epidermal growth factor, FGFs and TGFβs 26, 28, 29, 30, 31, 32, of which TGFβ and FGF are key mediators of EMT and are frequently and abundantly expressed in PCO tissues. Despite many studies on the activities and roles of TGFβ in lens, it remains unknown how TGFβs and FGF 2 synergistically act in EMT and how these molecules affect gene expressions including αSMA and tropomyosins (Tpms) during PCO development.

A number of target genes of FGF2 and TGFβ have been identified whose expression is activated by them, some of which are overly stimulated and implicated in EMT process including αSMA, fibronectin and Tpms, and thereby PCO formation 2, 33, 34, 35. More recently, our group identified modulation in expression of Tpms, specifically high molecular weight Tm isoforms from *Tpm1* and *Tpm2* genes, in a rodent model of PCO and in LECs obtained from cataractous humans of various ages 36. We previously reported expression of Tpm1/2 was minimal in rat LECs, and expression of Tpm1/2 that increased selectively during EMT was linked to fibrosis in PCO 36. Other cellular abnormalities, particularly in aberrant expression of cytoskeleton and ECM proteins, are induced because of overshooting of cellular signalling mediated by reactive oxygen species (ROS) 37. It is known that ROS‐induced damage to cells is related to ROS‐driven overstimulation TGF‐β1‐mediated signalling 38, 39 leading to over‐modulation of certain genes expression, including αSMA and TGF‐β‐induced protein (βig‐h3). Overexpression of those genes was involved in cataractogenesis, PCO and pathophysiological disorders of cells and tissues 31, 39, 40.

Previously, we showed that LECs deficient in peroxiredoxin 6 (Prdx6) display increased expression of ROS, phenotypic changes, a characteristic of terminal cell differentiation and EMT 39. Prdx6 provides cytoprotection against internal and external environmental stresses and plays a role in cellular signalling by detoxifying ROS thereby controlling gene regulation 39, 41, 42, 43. Using proteomic analysis of Prdx6‐deficient (*Prdx6* ^*−/−*^) mouse LECs, we found that such cells displayed elevated expression of cytoskeleton proteins Tpm1, Tpm2 and vimentin 44. Therefore, we posit that because Tpms are implicated in regulation of cellular activities by stabilizing ECM proteins (specifically actin microfilaments), aberrant expression of *Tpm1* and *Tpm2* genes is likely to be involved in the phenotypic alteration of *Prdx6* ^*−/−*^ LECs in mice.

Furthermore, Tpms are recognized as actin filament stabilizing proteins, regulating the dynamics and structural properties of the filaments by controlling the interaction of the filaments with actin‐binding proteins 45, 46, 47. The human tropomyosin genes should be known as *TPM1* through *TPM4* (*Tpm1* through *Tpm4* for mouse and rat tropomyosin) to be consistent with other gene nomenclatures 48. The various isoforms generated *via* alternative exon splicing are listed under each gene 47. The balance between levels of isoforms in a given cell determines the cell's Tpm functions 49, 50, 51, 52. Several TGFβ target genes, including *Tpm1*,* Tpm2*, α‐actinin1 and calponin2‐encoding actin‐binding proteins, were implicated in the assembly of stress fibres 51, 53, and Tpms played a crucial role in stabilizing actin filaments 54. TGFβ specifically up‐regulates expression of *Tpm1* and *Tpm2* genes but has no effect on regulation of *Tpm3* and *Tpm4* genes, which encode low molecular weight Tpms 51, 53. In addition, our group demonstrated an increased abundance of Tpm1/2 in differentiating LECs, suggesting involvement of TGFβ‐induced deleterious signalling in the induction of Tpm1/2 55. Given the above scenario, we surmised that over expression and activation of TGFβ induced by both surgical stress and ROS during cataract surgery induces and accelerates EMT by up‐regulating *Tpm1/2* genes, leading to PCO 55 and the process may be modulated by FGF2 in cellular microenvironment. In this study, we determined the role and potential of FGF2 to act as an antagonist of aberrant TGFβ signalling and its inducible genes/proteins such as *Tpm1/2* and αSMA that play a part in EMT process and stress fibre formation in mouse and human LECs. In addition, we show the involvement of FGF2‐mediated MAPK/ERK 1/2 signalling on TGF‐β2‐induced EMT. Loss of Tpms in TGFβ‐evoked EMT by FGF2 was significantly linked to MAPK/ERK1/2 pathway. We provide evidence for a regulatory role(s) of Tpms in the EMT process leading to PCO, and how the process may be restored by FGF2 in a concentration‐dependent manner. Understanding the underlying mechanisms of EMT, a cause of PCO, and its regulation by TGFβ, FGF2 and Tpms is important to develop new treatments to inhibit EMT and postpone PCO, secondary cataract.

## Materials and methods

### Cell culture

Primary cultured LECs were generated from 6W BalbC mice (*n* = 8) as described previously 56. Mice LECs (MLECs) were maintained in Dulbecco's modified Eagle's medium (DMEM; WAKO, Osaka, Japan) with 10% foetal bovine serum (FBS; Sigma‐Aldrich, St. Louis, MO, USA) at 37°C in an air/CO_2_ (19:1) atmosphere as described 39. Cells from 3 to 5 passages were used for the experiments. Simian virus 40‐transformed Huma LECs (HLECs) (SRA01/04) were kindly gifted by Dr. Nobuhiro Ibaraki (Ibaraki Eye Clinic, Tochigi, Japan). Human LECs were cultured in DMEM supplemented with 20% FBS.

To examine the effects of FGF2 (PEPROTECH, RockyHill, NJ, USA) and/or TGF‐β2 (HumanZyme, Chicago, IL, USA), MLECs or HLECs were plated in triplicate into 35 mm culture dishes (TPP^®^ Techno Plastic Products AG, Trasadingen, Switzerland). Cells growing in DMEM containing 0.1% bovine serum albumin (BSA) (WAKO) in the presence or absence of various test growth regulators received 0.001–10.0 ng/ml FGF‐2 or 0–10 ng/ml TGF‐β2 every other day for up to 4 days.

### RNA interference

siRNAs were transfected into cells according to the protocol recommended for Lipofectamine^®^ RNAi MAX reagent (ThermoFisher Scientific Japan Ltd., Tokyo, Japan). MLEC and HLECs were transiently transfected with siRNAs against a mixture of mouse, and human Tpm1 (Silencer^®^ Select pre‐designed siRNA, ID:s75390, ThermoFisher Scientific Japan Ltd.) and mouse and human Tpm2 (Silencer^®^ Select pre‐designed siRNA, ID:s75393) or negative control (Silencer^®^ Select Negative control#1 siRNA: NC‐siRNA). The final concentrations of the siRNAs were 10 nM.

### Western blot analysis

Protein lysates of MLECs or HLECs were prepared in ice‐cold radioimmune precipitation (RIPA) buffer, and SDS‐PAGE and protein blot analysis was performed as described 38, 57, 58. The membranes were probed with antimouse Tpm1/2 monoclonal antibody (Ab) (TM311) (Abcam^®^, Cambridge, MA, USA), antimouse αSMA monoclonal Ab (Sigma‐Aldrich), anti‐rabbit p44/42 MAPK (Erk1/2) monoclonal Ab (Cell Signaling Technology (CST) Japan, K.K., Tokyo, Japan), and anti‐rabbit phospho‐p44/42 MAPK (Erk1/2) monoclonal Ab (CST Japan). Anti‐rabbit glyceraldehyde‐3‐phosphate dehydrogenase (GAPDH) polyclonal Ab (Sigma‐Aldrich) was used to demonstrate that equal amounts of protein were loaded onto each lane.

### Real‐time reverse transcriptase‐polymerase chain reaction (RT‐PCR)

Total RNA from the MLECs or HLECs was extracted using an RNeasy Mini Kit (Qiagen, Valencia, CA, USA) per manufacturer's instructions. To measure the expression of mouse and human Tpm mRNAs, we conducted relative quantification of mRNA using a Prism7300 (Applied Biosystems^®^, ThermoFisher Scientific Japan Ltd., Tokyo Japan). PCR amplification was performed using a TaqMan Universal Master Mix and pre‐developed mouse Tpm2 probe mix (Applied Biosystems^®^), which recognize Tpm1 isoform, and mouse Tpm1 probe mix, which recognizes Tpm2, 3 and 5 isoforms. The relative quantity of Tpm1/2 and Tpm2 mRNA was determined using the comparative Ct method and then normalized using a pre‐developed TaqMan ribosomal RNA control reagent VIC probe as an endogenous control (Applied Biosystems^®^).

### ERK‐MAPK signalling pathway assay

The activities of ERK kinase were examined by Western blot analyses using antibodies against their phosphorylated forms. For the analysis of ERK activity, MLECs were treated with TGFβ2 (10 ng/ml) and/or FGF2 (10 ng/ml) for 10 and 60 min. To analyse the effect of a pharmacological inhibitor of FGF receptor (FGFR) (SU5402: SU, Sigma‐Aldrich) and a potent and selective inhibitor of MAP kinase (also known as MAPK/ERK kinase or MEK kinase) (PD98059: PD, Sigma‐Aldrich) on FGF2‐induced ERK phosphorylation, MLECs were pretreated with PD or SU with/without TGFβ2 (10 ng/ml) and/or FGF2 (10 ng/ml) for 60 min and 24 hrs.

### Immunofluorescence labelling and F‐actin staining

HLECs were grown on collagen‐coated eight‐well culture slides (Matsunami Glass Ind., Ltd., Osaka, Japan) and treated with the presence or absence of 10 ng/ml TGFβ2 and 10 ng/ml FGF2 in DMEM containing 2% FBS for 24 hrs. The cells were fixed with 4% paraformaldehyde and permeabilized with 0.05% Triton X‐100 (Sigma‐Aldrich). Tpm1/2 was stained with antimouse Tpm1/2 monoclonal antibody (Ab) (TM311) (Abcam^®^) and goat antimouse IgG (H+L) secondary Ab, Alexa Fluor^®^ 594 conjugate (ThermoFisher Scientific Japan Ltd.). F‐actin filaments were stained with CytoPainter phalloidin‐iFluor 488 reagent (Abcam^®^). The cell nucleus was stained with 4,6‐Diamidino‐2‐phenylindole, dihydrochloride (DAPI; Fluoroshield Mounting Medium with DAPI, ImmunoBioScience Corp., Mukilteo, WA, USA). Fluorescent images were captured using EVOS^®^ FLoid^®^ Cell Imaging Station (ThermoFisher Scientific Japan Ltd.).

### Cell migration assay

Cell migration assays were performed using Radius™ 24‐Well Cell Migration Assay Kit (Cell Biolabs, Inc., San Diego, CA, USA) per manufacturers’ protocol. Briefly, MLECs were grown on pre‐treated Radius™ 24‐well cell migration plate (Cell Biolabs, Inc.) at 2.0 × 10^5^/well for 9 hrs. Cells do not attach in the Radius™ gel spot area (~0.68 mm). Plates were treated with Radius™ gel removal solution to expose the cell‐free area to cell migration. After removal of gel, cells were treated with the presence or absence of 10.0 ng/ml FGF2 or 10 ng/ml TGFβ2 in DMEM containing 0.1% BSA for 24 hrs to observe their effect on cell migration.

### Statistical analysis

Data were reported as means ± S.D.s and analysed by one‐way anova, followed by a *t‐*test when appropriate, with *P* < 0.05 deemed significant.

## Results

### Effect of TGFβ and/or FGF2 on morphological changes and the expression of Tpm1/2 and αSMA, EMT markers in MLECs *in vitro*

**Figure 1:**
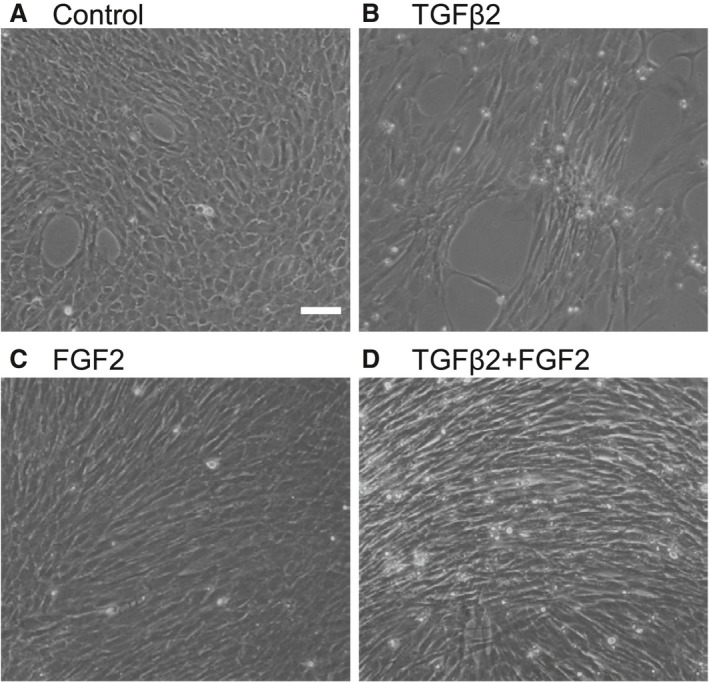
TGFβ2‐ and/or FGF2‐mediated phenotypic changes of LECs *in vitro*. Cultured MLECs were plated in 35 mm dishes at a density of 1 × 10^5^ in DMEM with 10%FBS for 24 hrs. LECs were treated with 10 ng/ml of TGFβ2 and/or FGF2 in DMEM containing 0.1% BSA for 2 days. Phase contrast photomicrographs were taken with a digital camera. Data were from three experiments. Scale bar, 90 μm.

To examine whether MLECs treated with TGFβ or TGFβ plus FGF2 showed phenotypic change, we selected TGFβ2 as the major isoform expressed in eyes, which plays a role in PCO 59, and FGF2 which is the most potent in FGF family. We performed phase contrast microscopic observation to analyse phenotypes of MLECs with/without addition of TGFβ2 (10 ng/ml) and/or FGF2 (10 ng/ml) for 48 hrs. Untreated MLECs were cuboidal shaped and organized in compact islets (Fig. 1A). After 48 hrs with TGF‐β, cells in these islets showed a spindle‐shaped morphology, elongated and underwent EMT‐like change (Fig. 1B) in response to TGFβ2 compared to untreated control (Fig. 1A). Many rounded dead cells were observed. FGF2‐treated MLECs were more proliferated than control (Fig. 1C). MLECs treated with TGFβ2 plus FGF2 (10 ng/ml each) were elongated showing fibroblastic‐like changes (Fig. 1D). These results prompted us to examine the contribution of TGFβ alone or in combination with FGF2 in mobilizing LECs towards EMT or vice versa.

**Figure 2:**
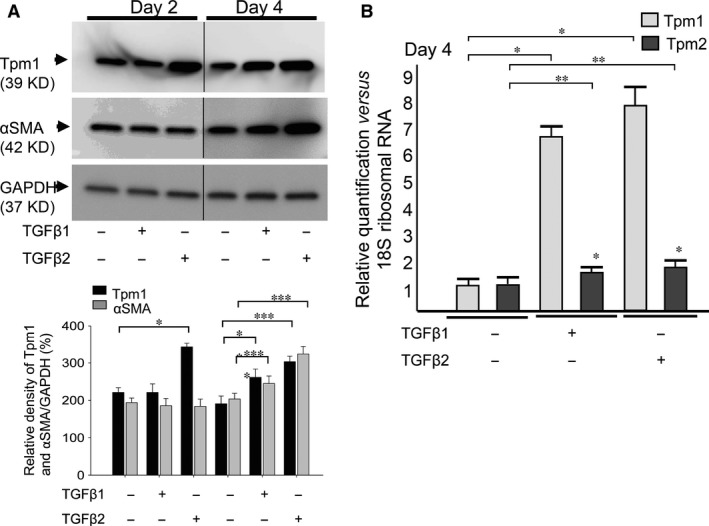
Expression of Tpm1/2 and αSMA in response to TGFβ1 and TGFβ2 in MLECs. Cultured MLECs were plated in 35 mm dishes at a density of 1 × 10^5^ in DMEM with 10%FBS for 24 hrs. LECs were treated with 10 ng/ml of TGFβ1 or TGFβ2 in DMEM containing 0.1% BSA for 2 days. (**A**) Cell lysates were prepared, and Western blotting analysis was performed. αSMA was used as the marker of EMT. GAPDH was used for control of protein concentration on Western blot analysis. Relative densities of Tpm1/2, αSMA and GAPDH were determined using the Image Quant LAS 4000 (GE Healthcare UK Ltd. Buckinghamshire, England). Data are representative of three experiments. (**B**) Total RNA was prepared, and real‐time PCR analysis was performed. 18s ribosomal RNA was used for control of cDNA concentration on real‐time PCR analysis. Relative quantity of Tpm1/2 was determined using Prism 7300 System SDS RQ Study Software (Applied Biosystems^®^). Data were from three experiments and were reported as means ± S.D.s.

**Figure 3:**
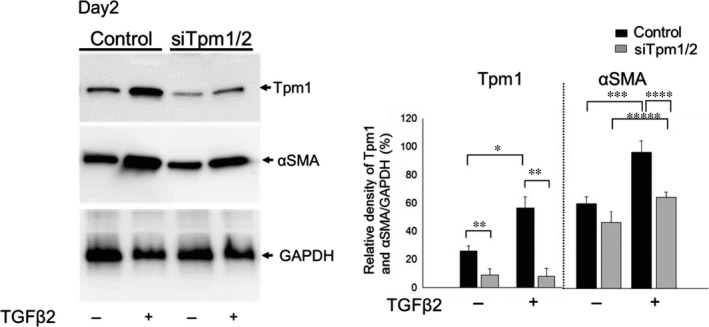
Expression of Tpm1/2 and αSMA in response to TGFβ2 in MLECs transfected with siRNA against Tpm1/2 and negative control. Cultured MLECs were plated in 35 mm dishes at a density of 1 × 10^5^ in DMEM with 10%FBS for 24 hrs. MLECs were transfected with siRNA against Tpm1/2 and negative control. At 24 hrs after transfection, LECs were treated with 10 ng/ml of TGFβ2 in DMEM containing 2%FBS for 2 days. A: Cell lysates were prepared, and Western blotting analysis was performed. αSMA was used as the marker of EMT. GAPDH was used for control of protein concentration on Western blot analysis. Data were from three experiments and were reported as means ± S.D.s.

Figure 1 shows TGFβ2, and TGFβ2 in combination with FGF2 differentially affected LECs phenotype. Next we examined levels of genes which have been reported to be involved in regulation of shape and geometric features of cells including αSMA and Tpms. Western blotting analysis of levels of αSMA and Tpm1/2 protein in MLECs in response to TGFβ1 and β2 (10 ng/ml, each) using antibody specific to αSMA and Tpm1/2 revealed that TGFβ1‐treated cells displayed enhanced expression of Tpm1 and αSMA proteins on day 4 (Fig. 2A; **P < 0.016, ****P < 0.05). Similarly, TGFβ2‐treated MLECs showed increased abundance of Tpm1 and αSMA proteins (Fig. 2A; *P < 0.0002, ***P < 0.0015, Fig. 3A and B; *P < 0.0042, ***P < 0.0028). TGFβ2 was more potent in up‐regulating expression. To determine whether increased expression of αSMA and Tpm1 by TGFβ was transcriptional level, we performed real‐time RT‐PCR. We found the expressions of Tpm1/2 mRNA were significantly elevated in MLECs following TGFβ1 and TGFβ2 treatment (Fig. 2B, *P < 0.0001; **P < 0.05). Further, levels of Tpm1/2 protein in Tpm1/2 siRNA‐transfected MLECs were reduced after treatment with/without TGFβ2 on day 2 (Fig. 3A and B; **P < 0.0061). TGFβ2‐treated MLECs showed increased abundance of Tpm1 protein after transfection with NC‐siRNA (control) (Fig. 3A and B; *P < 0.0042). Similarly, TGFβ2‐treated MLECs showed increased abundance of αSMA protein after transfection with NC‐siRNA (control) and siRNA against Tpm1/2 (siTpm1/2) (Fig. 3A and B; ***P < 0.0028, ****P < 0.0241). Levels of αSMA protein in TGFβ2‐treated MLECs were reduced in siTpm1/2 group in comparison with control group suggesting EMT induced by TGFβ2 may be inhibited by Tpm1/2 knock‐down (Fig. 3A and B; ****P < 0.0024).

**Figure 4:**
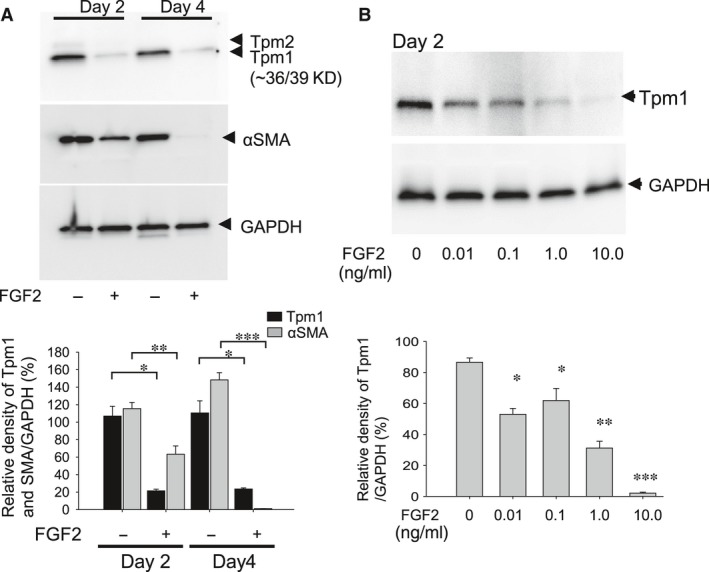
Expression of Tpm1 and αSMA in MLECs in response to FGF2. Cultured MLECs were plated in 35 mm dishes at a density of 1 × 10^5^ in DMEM with 10%FBS for 24 hrs. (**A**) MLECs were treated with 0 or 10 ng/ml of FGF2 in DMEM containing 0.1% BSA for 2 days. αSMA was used as the marker of EMT. (**B**) MLECs were treated with 0, 0.01, 0.1, 1.0 or 10 ng/ml of FGF2 in DMEM containing 0.1% BSA for 2 days. **A** and **B**: Cell lysates were prepared, and Western blotting analysis was performed, with GAPDH used for control of protein concentration. Data were from three experiments and were reported as means ± S.D.s.

FGF2‐treated cells also exhibited changes as shown in Figure 1. MLECs treated with FGF2 for 2 and 4 days showed a marked reduction in the expressions of Tpm1 and αSMA protein (Fig. 4A; *P < 0.0004, **P < 0.002, *** *P* < 0.00001). To investigate the effect of FGF2 concentration on levels of Tpm1 or αSMA expression in MLECs, cells were treated with different concentrations of FGF2 for 2 days. We found that reduction of Tpm1 expression in MLECs was inversely related to the concentration of FGF2 (Fig. 4B; *P < 0.007, **P < 0.0005, ***P < 0.0000001). Unlike TGFβ, FGF2 suppressed the expression of Tpm1 and αSMA in MLECs suggesting that FGF2 acts as an antagonist of TGFβ signalling.

**Figure 5:**
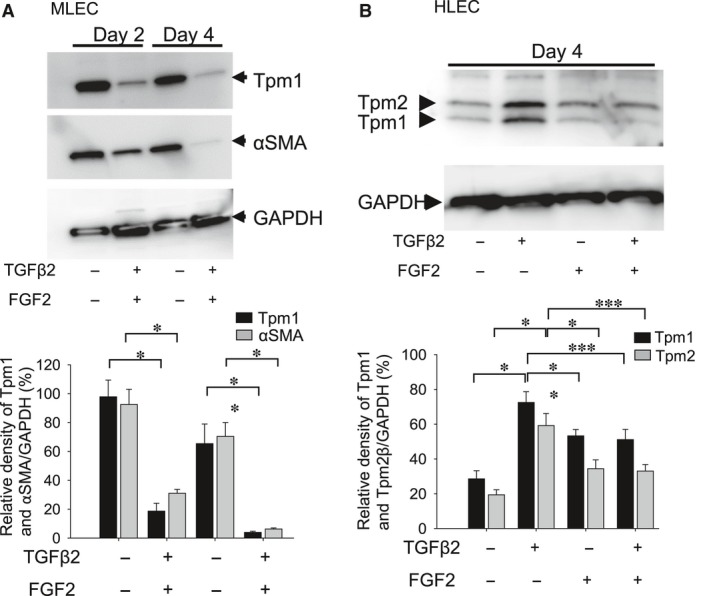
Expression of Tpm1 and αSMA proteins in MLECs and HLECs stimulated with FGF2 and TGFβ2. Cultured MLECs (**A**) and HLECs (**B**) were plated in 35 mm dishes at a density of 1 × 10^5^ in DMEM with 10%FBS for 24 hrs. (**A**) MLECs were treated with 0 or 10 ng/ml of TGFβ2 and FGF2 in DMEM containing 0.1% BSA for 2 and 4 days. αSMA was used for marker of EMT. (**B**) HLECs were treated with 0 or 10 ng/ml of TGFβ2 and/or FGF2 in DMEM containing 0.1% BSA for 4 days. **A** and **B**: Cell lysates were prepared, and Western blotting analysis was performed, with GAPDH used for control of protein concentration. Data were from three experiments and were reported as means ± S.D.s.

Next, to examine the combined effect of TGFβ2 and FGF2 on Tpm1 and αSMA expression in MLECs, cells were stimulated with FGF2 (10 ng/ml) and TGFβ2 (10 ng/ml) for 2 and 4 days. Based on the observed EMT‐like changes in MLECs (Fig. 1D), we speculated that FGF2 and TGFβ2 may induce Tpm1 or Tpm2 expression. However, FGF2 showed an opposing action against TGFβ2′s adverse effects, with significantly reduced expression of Tpm1 and αSMA proteins (Fig. 5A; *P < 0.0006, **P < 0.002). Similarly, in HLECs treated with TGFβ2 and FGF2 in combination, the expression of Tpm1 and Tpm2 protein was increased in response to TGFβ2 and decreased in response to FGF2 and TGFβ2 at day 4 (Fig 5B; *P* < 0.0008, **P < 0.013, ***P < 0.005). Taken together, these findings indicate that TGFβ2 induces expression of Tpm1, Tpm2 and αSMA, and in contrast, FGF2 acts as antagonist and reverses the TGFβ2‐mediated aberrant activation of Tpm1, Tpm2 and αSMA expression in MLECs.

### Effect of TGFβ2 and/or FGF2 on stress fibre formation in Tpm1/2‐siRNA‐transfected HLECs

**Figure 6:**
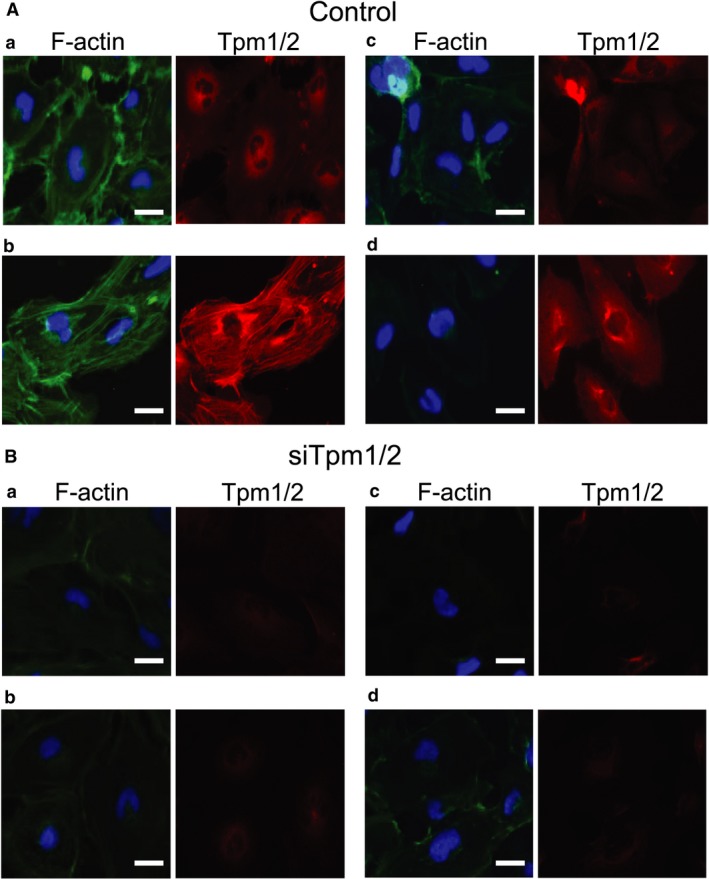
F‐actin staining with phalloidin in HLECs stimulated with 10 ng/ml TGFβ2 and/or 10 ng/ml FGF2. Cultured HLECs were plated in collagen‐coated eight‐well chamber slides at a density of 4 × 10^4^ in DMEM with 10%FBS for 24 hrs. MLECs were transfected with siRNA against Tpm1/2 (**A**) and negative control (**B**). At 24 hrs after transfection, LECs were treated with 10 ng/ml of TGFβ2 and/or FGF2 in DMEM containing 2% FBS for 24 hrs. To observe immunolocalization of Tpm1/2 and stress fibre formation, immunolabelling of Tpm1/2 (**A** and **B**) and co‐labelling of F‐actin (**A** and **B**) were performed in HLEC treated without TGFβ2 (**A**‐a and **B**‐a), with TGFβ2 (**A**‐b and **B**‐b), with FGF2 (**A**‐c and **B**‐c) or TGFβ2 + FGF2 (**A**‐d and **B**‐d) treatment for 2 days. The cell nucleus was stained with DAPI (**A** and **B**; Blue colour). Data are representative of three experiments. Scale bar, 15 μm.

The actin cytoskeleton plays a crucial role in regulation of cellular processes including proliferation, apoptosis, cell migration and invasion 54, 60. TGFβ induces rapid reorganization of the actin cytoskeleton, whereas prolonged incubation with TGFβ induces stress fibres 61, 62. Stress fibres, which are contractile bundles of actin filaments and actomyosin, are essential for cell adhesion, migration and maintenance of cell shape 63. To examine stress fibre formation in HLECs, we performed F‐actin staining with phalloidin as described in the ‘Materials and Methods’ section. Microscopic examination revealed an absence of stress fibres (Fig. 6A‐a, left) in untreated HLECs, and Tpm1/2 was faintly immunolabelled in the perinuclear area of untreated HLECs (Fig. 6A‐a, right). In contrast, after 48 hrs with TGF‐β, F‐actin was assembled into thick parallel bundles, or actin stress fibres, traversing the ventral cell surface (Fig. 6A‐b, left) and Tpm1/2 was localized at these stress fibres indicating involvement of TGFβ2 in their formation (Fig. 6A‐b, right). In HLECs treated with FGF2, stress fibre formation was markedly reduced (Fig. 6A‐c, left) and levels of Tpm1/2 were reduced. In HLECs treated with TGFβ2 and FGF2, stress fibre formation was reduced and located in the cellular periphery (Fig. 6A‐d, left) and Tpm1/2 was immunolabelled in the perinuclear area of HLECs. This result suggests that stress fibre formation in TGFβ2‐treated HLECs was blocked because of the presence of FGF2. Further, siRNA against Tpm1/2 was transfected in HLECs. Immunolabelling of Tpm1/2 was reduced in Tpm1/2‐siRNA‐transfected HLECs (Fig. 6B a–d, right). In Tpm1/2‐siRNA‐treated HLECs, stress fibres were not induced after treatment with or without TGFβ2 and/or FGF2 (Fig. 6B b–d, right). These results suggest that FGF2 and knock‐down of Tpm1/2 may inhibit TGFβ2‐mediated stress fibre formation in LECs. Higher expression of Tpm1/2 may induce stress fibre formation in LECs.

### Effect of TGFβ2 and/or FGF2 on cell migration in MLECs

**Figure 7:**
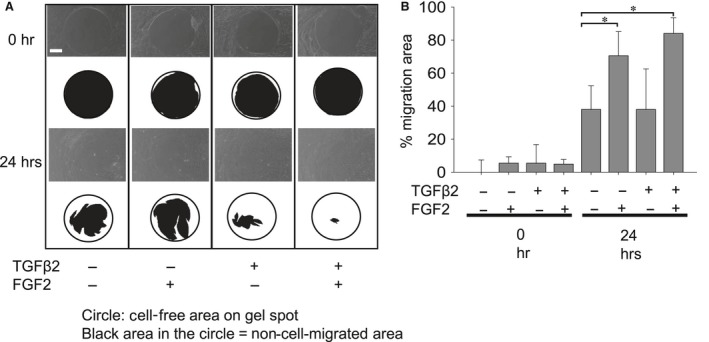
Migration of MLECs treated with/without TGFβ2 and/or FGF2. MLECs were plated in 24‐well plates pre‐coated with collagen type I, at a density of 1 × 10^5^ in DMEM with 10%FBS for 24 hrs. Each plate contained 0.68 mm non‐toxic biocompatible hydrogel spot (Radius™ Gel) where cells cannot attach. After hydrogel removal to expose the cell‐free region, MLECs were treated with 0 or 10 ng/ml of TGFβ2 and/or FGF2 in DMEM containing 0.1% BSA for 24 hrs. Phase contrast micrographs were then taken with a digital camera. Data shown are representative of three experiments. The cell‐free area was analysed using MultiGauge Software (Fuji Film, Tokyo, Japan). Data were from three experiments and were reported as means ± S.D.s. Scale bar, 180 μm.

To determine the effect of TGFβ2 with/without FGF2 on migration, we conducted a cell migration assay as described in the ‘Materials and Methods’ section. We found that treatment with TGFβ2 induced MLECs migration (Fig. 7A and B; *P < 0.01), which was further augmented, when MLECs were treated with both TGFβ2 and FGF2 in combination (Fig. 7A and B; *P < 0.01), indicating TGFβ2 in combination with FGF2 accelerated the migration process of MLECs. Further, treatment with FGF2 alone did not induce the migration of MLECs (Fig. 7A and B).

### FGF2 inhibits TGFβ2‐induced Tpm1 and αSMA gene, but promotes TGFβ2‐induced cell migration *via* activation of MAPK/ERK pathway

**Figure 8:**
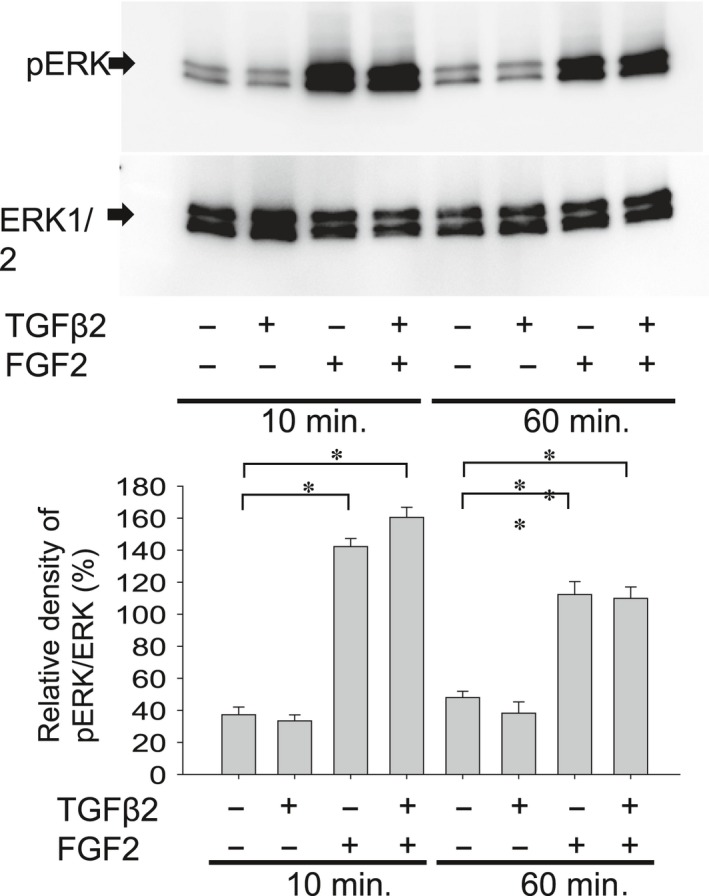
Effect of FGF2 and/or TGFβ2 stimulation on activation of ERK1/2 pathway in MLECs. To evaluate the effect of FGF2 with/without TGFβ2 stimulation on phosphorylation of ERK1/2 in MLECs, MLECs were treated with 0 or 10 ng/ml of TGFβ2 and/or FGF2 in DMEM containing 0.1% BSA for 10 or 60 min. Cell lysates were prepared, and Western blotting analysis was performed using anti‐rabbit p44/42 MAPK (Erk1/2) monoclonal Ab or anti‐phospho‐rabbit p44/42 MAPK (Erk1/2) monoclonal Ab. Data were from three experiments and were reported as means ± S.D.s.

**Figure 9:**
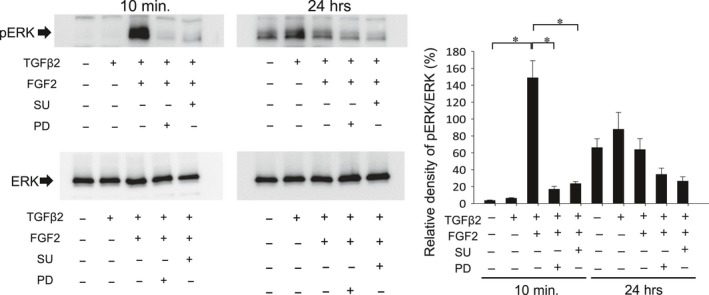
Effect of FGFR antagonist (SU) and MECK inhibitor (PD) on FGF2‐induced activation of ERK pathway. MECK inhibitor (PD) to block ERK pathway and FGFR antagonist (SU) to inhibit FGF2 stimulation were used. To evaluate the effect of FGF2 with/without TGFβ2 stimulation on phosphorylation of ERK1/2, MLECs were treated with 0 or 10 ng/ml of TGFβ2 and/or FGF2 in DMEM containing 0.1% BSA with/without SU or PD for 10 min or 24 hrs. Cell lysates were prepared, and Western blotting analysis was performed using anti‐rabbit p44/42 MAPK (Erk1/2) monoclonal Ab or anti‐phospho‐rabbit p44/42 MAPK (Erk1/2) monoclonal Ab. Data were from three experiments and were reported as means ± S.D.s.

We next examined the involvement of signalling pathway(s) responsible for FGF2‐mediated down‐regulation of Tmp1α expression and promotion of cell migration in the presence or absence of FGF2 with TGFβ2. To investigate the effect of FGF2 with/without TGFβ2 stimulation on phosphorylation of ERK1/2 in MLECs, MLECs were treated with FGF2 and/or TGFβ2. Cellular extracts were immunoblotted using antibody as indicated in Fig. 8. FGF2 induced the ERK phosphorylation within/at 10 min and remained for 60 min with/without TGFβ2 stimulation. These results suggest that FGF2 activated ERK pathway, as it stimulated ERK phosphorylation, and wherein treatment of TGFβ2 had no effect on ERK phosphorylation (Fig. 8; *P < 0.00002, **P < 0.0003). For validation, we used MECK inhibitor (PD) to block the ERK pathway, and FGFR antagonist (SU) to inhibit FGF2 stimulation. As shown in Fig. 9, FGF2‐stimulated phosphorylation of ERK was inhibited by PD and SU at 60 min (*P < 0.0005) confirming the existence of ERK pathway in MLECs, which was activated by FGF2.

**Figure 10:**
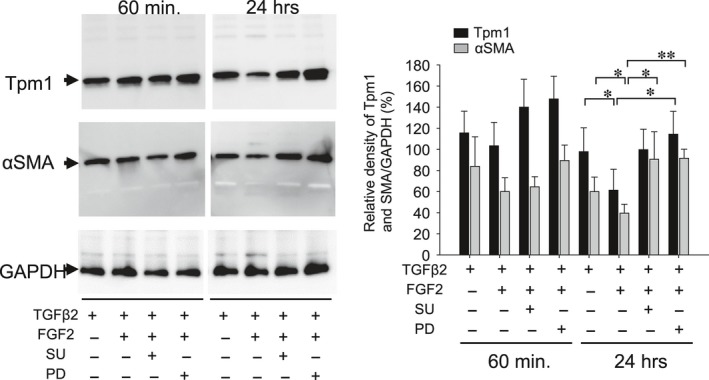
Effect of MECK inhibitor (PD) and FGF2 antagonist (SU) on repression of Tpm1 and αSMA expression in response to FGF2. Cultured MLECs were plated in 35 mm dishes at a density of 1 × 10^5^ in DMEM with 10%FBS for 24 hrs. MLECs were treated with 0 or 10 ng/ml of TGFβ2 plus FGF2 in DMEM containing 0.1% BSA with/without SU or PD for 60 min and 24 hrs. Cell lysates were prepared, and Western blotting analysis was performed using anti‐Tpm1/2 Ab and anti αSMA Ab, with GAPDH used for control of protein concentration. Data were from three experiments and were reported as means ± S.D.s.

MAPK has been reported to be involved in regulation of Tpms 64. To investigate the MAPK/ERK pathway involved in the repression of Tpm gene, we tested the effects of MECK inhibitor and FGF2 antagonist on FGF2‐induced repression of Tpm1 and αSMA expression (Fig. 10). We observed PD inhibited the repression of Tpm1 induced by FGF2 at 24 hrs of treatment (Fig. 10; *P < 0.05), and both PD and SU inhibited the repression of αSMA expression induced by FGF2 at 24 hrs of treatment (Fig. 10; *P < 0.05, **P < 0.002).

**Figure 11:**
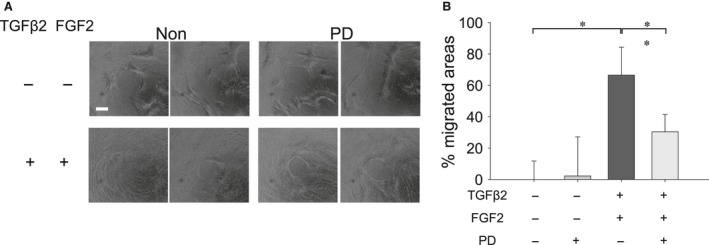
Effect of MECK inhibitor (PD) on migration of MLECs treated with TGFβ2 and FGF2. MLECs were plated in 24‐well plates, pre‐coated with collagen type I, at a density of 1 × 10^5^ in DMEM with 10%FBS for 24 hrs. After hydrogel removal to expose the cell‐free region, MLECs were treated with 0 or 10 ng/ml of TGFβ2 plus FGF2 with/without PD in DMEM containing 0.1% BSA for 24 hrs. Phase contrast micrographs were then taken with a digital camera. Data were from three experiments and were reported as means ± S.D.s. Scale bar, 180 μm.

To determine whether MAPK/ERK pathway was involved in the FGF2 and TGFβ2‐induced cell migration, we examined the effect of PD on migration of MLECs treated with TGFβ2 and FGF2. Cell migration was induced by treatment with TGFβ2 and FGF2, and PD inhibited such migration (Fig. 11; *P < 0.02, **P < 0.05) suggesting that both factors are functionally involved in LECs’ migration.

## Discussion

This study provides evidence that Tpm1/2 plays an important role in TGFβ2‐induced EMT, stress fibre formation and cell migration in LECs. Knock‐down of Tpm1/2 by siRNA blocks the elevation of αSMA and formation of actin stress fibre. The process of EMT is implicated in cancer progression, wound healing and tissue fibrosis as well as normal embryonic development 65, 66, 67 (Thiery, 2003; Lee *et al*., 2006)^68^. In cancer, EMT leads to generation of more aggressive and invasive carcinoma cells as well as cancer stem cells 68. EMT involves disassembly of the polarized epithelial architecture and remodelling of the cell cytoskeleton, including intermediate and actin filaments. The present study revealed a dynamic process in the initiation and progression of EMT that occurs in PCO. We demonstrated that the effect of aberrant TGFβ2 signalling on LECs migration, proliferation, stress fibre and EMT is influenced by FGF2 growth factor in the cellular microenvironment. Undoubtedly, this effect of FGF2 can be associated with its concentration. Interestingly, we found that FGF2 and knock‐down of Tpm1/2 antagonize the effect of TGFβ2 on EMT features (Figs 2, 5 and 6) by repressing the expression of Tpms, αSMA and stress fibres. In contrast, this molecule synergistically acted with TGFβ2 and promoted cell migration (Fig. 7). This function of FGF2 may be related to FGF2 concentration in the cellular microenvironment. We posit that during the development of EMT, FGF2 and TGFβ2 differentially affect the EMT process in a manner linked to their differential concentrations. Tpms are actin‐stabilizing proteins that play a major role in maintaining cellular integrity. By assessing expression of *Tpm1* and *Tpm2* genes, in MLECs and HLECs, we found that growth and differentiation of LECs were differentially regulated by TGFβ and FGF2 according to differential expression of Tpm genes (Figs 2–5, 7). TGFβ induces epithelial to myofibroblastic transition (EMyoT) which was accompanied with Tpm and αSMA expression (a maker for EMT). Importantly, Tpms may promote the formation of stress fibres undergoing EMyoT which expresses F‐actin. However, we found FGF2 suppresses the TGFβ2‐induced up‐regulation of Tpm and αSMA (Fig. 5). Reduction of Tpm by FGF2 suppresses the formation of stress fibres and thereby activates fibroblastic LECs which induce cell migration as we observed (Figs 1 and 6). This result indicates that LECs in PCO that contain abundant FGF2 are less differentiated than those expressing predominant TGFβ2 in the cellular microenvironment.

We previously reported increased expression of Tpm1/2 during EMT and demonstrated that selective elevation of Tpm1/2 in rat LECs was correlated with fibrosis observed in PCO using *in vivo* rat model 55. In addition, we showed that expression of Tpm1/2 was induced/elevated in transdifferentiated multi‐layered and spindle‐shaped LECs in a rat model of PCO and human cataracts with anterior subcapsular cataract including in differentiated HLECs in a dislocated lens capsule 55. Data from these previous results strongly suggested that expression of Tpm1/2 is linked with progression of PCO 55. Several TGF‐β target genes, including *Tpm1* and *Tpm2*, have been implicated in the assembly of stress fibres 51, 53. Of these, Tpms in particular have been shown to play a crucial role in stabilizing actin filaments 54. Furthermore, we have also reported up‐regulation of Tpm1/2 expression in differentiating LECs (in the presence of TGFβ), demonstrating involvement of TGFβ‐induced deleterious signalling in the induction of Tpm1/2. In the past, several *in vitro* and *in vivo* studies have examined the role of TGFβ2 in EMT and wound healing processes using LECs 5, 6, 69. Transdifferentiation analogous to that observed in PCO can be induced by exposing LECs to TGFβ 5, 6, 70, 71.

Moreover, a major contribution of FGF2 in PCO development has been reported 1, 27, 72. FGF2 is a potent mitogenic growth factor which is present in the eye lens environment 27, and concentration of FGF2 may increase after cataract surgery 72. For instance, Wallentin *et al*. 73 showed that aqueous FGF2 was increased in rabbits up to 30 days following cataract extraction. Furthermore, in a study using a rabbit PCO model, the level of active TGFβ decreased and increased FGF2 stimulated cell proliferation, immediately after surgery, and at around 2 weeks after surgery, active TGFβ returned to normal level stimulating EMT 35. TGFβ2 has been shown to inhibit the proliferative effect of FGF2 on rabbit LECs growth 73. FGF2 reduced the contraction of a collagen gel by bovine LECs and the proportion of cells expressing αSMA 29, indicating that FGF2 has opposing actions to TGFβ2. Furthermore, simultaneous treatment of rat LECs explants with TGFβ2 and FGF2 were more affected by TGFβ2 than corresponding high cell coverage explants, showing greater cell loss, more marked formation of spindle cells and expression of αSMA 71. Excessive deposition of ECM and formation of plaques of swollen cells, also features of PCO 1, 71, occurred only when TGFβ was supplemented with FGF2 70, 71.

Our study found cotreatment with TGFβ2‐ and FGF2‐induced spindle‐shaped fibroblastic formation of HLECs and accelerated the EMT process. Furthermore, we found that TGFβ2 and FGF2 in combination suppressed the formation of stress fibres in LECs and the expression of Tpm and αSMA. Importantly, Tpm1/2 knock‐down inhibited the TGFβ2‐induced formation of stress fibres. Tpms have been shown to stabilize actin filaments 54. Thus, our study revealed that Tpms may induce stress fibre in response to TGFβ2 in the EMT process.

We found that TGFβ2 induced differentiation of LECs into two types of mesenchymal cells: one was Tpm1‐ and αSMA‐positive myofibroblastic cells generated through EMyoT by TGFβ2 alone, and the other was activated Tpm1‐ and αSMA‐negative fibroblastic cells generated through EMT by both TGFβ2 and FGF2 in combination. Furthermore, our result also indicated that TGFβ2 enhanced cell migration compared with non‐treated cells, and the co‐addition of FGF2 further promoted cell migration (Fig. 7). Based on these data, we believe that Tpm abundance may inhibit cell migration and reduced level of Tpm may induce cell migration as we showed that FGF2 markedly suppressed the expression of Tpms in LECs.

Moreover, FGF has been shown to be a major activator of the ERK‐MAPK pathway in the eye lens *in vivo* 74, 75. Further, FGF2 activation of ERK1/2 signalling could influence TGFβ2 induction of Tpm1 gene expression. The present study showed the inducible expression of Tpm1 was attenuated in the presence of PD, a specific inhibitor of MAPK/ERK signalling, and SU, an inhibitor of FGFR2. Also, cell migration induced by TGFβ2 and FGF2 was inhibited in the presence of PD. These data support the notion that ERK signalling affects cell migration and the formation of stress fibres by suppressing the expression of Tpm.

In summary, we demonstrated both independent and combined roles of TGFβ2 and FGF2 in the differential regulation of EMT and how this process is associated with Tpm expression including the implications for PCO development. Importantly, we found that FGF2 acts as an antagonist against TGFβ‐mediated EMT progression. We demonstrated that FGF2‐induced ERK signalling attenuates aberrant expression of Tpm1 and cell migration. Our results provide a novel insight into the regulation and function of Tpms during LEC differentiation, which is influenced profoundly by growth factors. These findings reveal that opposing effects of FGF2 and TGFβ2 on Tpm1/2 gene expression control the phenotypic plasticity of LECs on PCO progression. Further, Tpm1/2 may regulate other eye diseases such as pterygium and glaucoma and wound healing processes related to EMT.

We hope this study provides clues to develop new therapies of PCO and other EMT‐related diseases targeting the balance regulation of Tpms by growth factors. However, further in‐depth studies will be required to fully clarify the underlying mechanism of Tpm1/2 involvement in EMT process.

## Conflict of interest

This study was partly funded by Ono Pharmaceutical Co. Ltd (Osaka Japan).
